# Sex-Biased Expression of Olfaction-Related Genes in the Antennae of *Apis cerana* (Hymenoptera: Apidae)

**DOI:** 10.3390/genes13101771

**Published:** 2022-09-30

**Authors:** Hanchao Du, Wenting Su, Jiaxing Huang, Guiling Ding

**Affiliations:** 1 Key Laboratory of Pollinating Insect Biology of the Ministry of Agriculture and Rural Affairs, Institute of Apicultural Research, Chinese Academy of Agricultural Sciences, Beijing 100093, China; 2 College of Animal Science and Veterinary Medicine, Shanxi Agricultural University, Taigu, Jinzhong 030801, China

**Keywords:** *Apis cerana*, antennal transcriptome, olfaction-related genes, workers, drones

## Abstract

The olfactory system is essential for honeybees to adapt to complex and ever-changing environments and maintain cohesiveness. The Eastern honeybee *Apis cerana* is native to Asia and has a long history of managed beekeeping in China. In this study, we analysed the antennal transcriptomes of *A. cerana* workers and drones using Illumina sequencing. A total of 5262 differentially expressed genes (DEGs) (fold change > 2) were identified between these two castes, with 2359 upregulated and 2903 downregulated in drones compared with workers. We identified 242 candidate olfaction-related genes, including 15 odourant-binding proteins (OBPs), 5 chemosensory proteins (CSPs), 110 odourant receptors (ORs), 9 gustatory receptors (GRs), 8 ionotropic receptors (IRs), 2 sensory neuron membrane proteins (SNMPs) and 93 putative odourant-degrading enzymes (ODEs). More olfaction-related genes have worker-biased expression than drone-biased expression, with 26 genes being highly expressed in workers’ antennae and only 8 genes being highly expressed in drones’ antennae (FPKM > 30). Using real-time quantitative PCR (RT-qPCR), we verified the reliability of differential genes inferred by transcriptomics and compared the expression profiles of 6 ORs (*AcOR10*, *AcOR11*, *AcOR13*, *AcOR18*, *AcOR79* and *AcOR170*) between workers and drones. These ORs were expressed at significantly higher levels in the antennae than in other tissues (*p* < 0.01). There were clear variations in the expression levels of all 6 ORs between differently aged workers and drones. The relative expression levels of *AcOR10*, *AcOR11*, *AcOR13*, *AcOR18* and *AcOR79* reached a high peak in 15-day-old drones. These results will contribute to future research on the olfaction mechanism of *A. cerana* and will help to better reveal the odourant reception variations between different biological castes of honeybees.

## 1. Introduction

Highly developed chemosensory systems help insects adapt to complex and ever-changing environments [1,2]. As an important chemosensory type, olfaction plays a key role in insect behaviour, including foraging, mating, oviposition, communication and enemy avoidance [3]. Odourants reach the sensillar lymph by penetrating the cuticle through the pores at the surface of the sensillum [4]. The sensillar is rich in proteins, including odourant-binding proteins (OBPs), chemosensory proteins (CSPs), odourant receptors (ORs), gustatory receptors (GRs), ionotropic receptors (IRs), sensory neuron membrane proteins (SNMPs) and odourant-degrading enzymes (ODEs), which are the main proteins involved in the odourant reception process [4,5,6].

A honeybee colony normally consists of a fertile queen, hundreds of drones and thousands of sterile workers. Workers carry out numerous tasks, including foraging, defence, brood care, and cleaning, while drones specialize in mating [7]. In such a massive, complex and crowded collective, the olfactory system plays a critical role in honeybee communication. In honeybees, odourant reception takes place mainly in the antennae, the olfactory organs covered with hair-like structures called olfactory sensilla. The worker antennae consist of 10 flagellum segments, and the drone antennae consist of 11 flagellum segments [8]. There are approximately 6500 and 18,600 sensilla per antenna in the workers and drones, respectively [9,10]. The morphological differences between the antennae of workers and drones correspond to their different biological functions.

The eastern honeybee *Apis cerana* has a large native range in Asia and has been raised in China for thousands of years [11,12]. Previous studies have identified and characterized the expression of OBPs, CSPs, ORs, GRs, IRs and SNMPs at the gene level among workers [13,14,15,16] and compared the expression differences of OBPs and CSPs at the protein level between workers and drones of *Apis mellifera* [8]. However, a complete understanding of olfaction-related gene differences between *A. cerana* drones and workers is lacking. In this study, we performed transcriptome sequencing analysis on *A. cerana* antennae to identify putative olfaction-related genes (OBPs, CSPs, ORs, IRs, GRs, SNMPs and ODEs) and compared differentially expressed genes (DEGs) between workers and drones using real-time quantitative PCR (RT-qPCR). These results contribute to our knowledge of olfactory functions and to a better understanding of the functional variations between different biological castes of honeybees.

## 2. Materials and Methods

### 2.1. Sample Collection

*A. cerana* colonies were reared in Beijing, China. For transcriptome sequencing analysis, we captured foraging worker bees and mature drones that were leaving for flight at the entrance of the hive in the afternoon. Thirty pairs of antennae were collected and pooled to form each biological replicate.

To further explore the tissue- and age-specific expression patterns of selected ORs, we also collected different tissues from differently aged workers and drones. Capped brood combs close to eclosion were selected from three different healthy colonies and maintained in an incubator at 35 °C and 80% relative humidity. We marked the adult workers and drones on their thorax after emergence and returned them to the original colonies until sampling. The workers and drones were collected at 1, 5, 10, 15, 20, 25 and 30 days of age. Pairs of antennae were collected from 10 bees and pooled as one sample. For the 1- and 15-day-old drones and workers, other tissues, including the head (without antennae), thorax, abdomen, legs and wings, from 10 individuals per colony were also pooled together as one sample. All the samples were excised on dry ice and immediately placed in liquid nitrogen for storage until RNA extraction. Three biological replicates and three technical replicates were prepared for each group in all experiments.

### 2.2. RNA Extraction and RNA-Seq Library Sequencing

All samples were homogenized using a TissueLyser-24 (Jingxin, Shanghai, China) for 45 s (3×) at 70 Hz with two 2 mm stainless steel beads. Total RNA was extracted using TRIzol Reagent (Thermo Fisher Scientific, Waltham, MA, USA) in accordance with the manufacturer’s instructions. The purity, concentration and integrity of the RNA were assessed using a NanoDrop 2000 (Thermo Fisher Scientific, Waltham, MA, USA), Qubit 2.0 (Invitrogen, Carlsbad, CA, USA) and Agilent 4200 (Agilent Technologies Inc., Santa Clara, CA, USA), respectively.

The cDNA libraries were prepared using the VAHTS mRNA-seq v2 Library Prep Kit for Illumina (Vazyme Biotech Co., Nanjing, China), and Illumina sequencing was performed by Berry Genomics Co., Ltd. (Beijing, China), using the Illumina NovaSeq platform. For each sample, mRNA was purified from 2 µg of total RNA using poly-T oligo-attached magnetic beads and fragmented into small pieces using divalent cations at 94 °C for 5 min with fragmentation buffer. First-strand cDNA was synthesized using N6 random primers, followed by second-strand cDNA synthesis using RNaseH and DNA polymerase I. The cDNA then underwent an end repair process, adenylation of the 3′ ends and subsequent ligation of the adapter. The adaptor-ligated libraries were purified using VAHTS DNA Clean Beads (Vazyme Biotech Co., Nanjing, China) and enriched by PCR to create the final cDNA library. Then, the libraries were sequenced on the Illumina NovaSeq platform to generate 150 bp paired-end reads.

### 2.3. Differential Gene Expression Analysis

The sequencing adaptors and low-quality regions were trimmed from raw reads using Trimmomatic v0.6.6 [17]. The quality of the raw reads and clean reads was assessed by FastQC v2.10 [18]. The clean reads were mapped to the *A. cerana* genome (ftp://ftp.ncbi.nlm.nih.gov/genomes/refseq/invertebrate/Apis_cerana/latest_assembly_versions/GCF_001442555.1_ACSNU-2.0/; accessed on 6 December 2021) with HISAT2 v2.2.0 [19] and assembled with StringTie v2.1.7 [20].

The gene expression levels were acquired by the ballgown package v2.22.0 [21]. The expression level of genes was normalized to fragments per kilobase of transcript per million mapped fragments (FPKM) values. The repeatability of samples was evaluated by calculating the Pearson’s correlation coefficient for gene expression levels between all pairs of samples using the corrplot package v0.92 [22]. The Benjamini-Hochberg procedure was used for multiple-testing corrections [23]. Genes with an adjusted *p* value < 0.05 and |log_2_ (fold change)| > 1 were considered differentially expressed.

We performed Gene Ontology (GO) enrichment and KEGG pathway analyses of the DEGs using the cluster Profiler package v.3.18.1 [24]. Data visualization and plotting were performed using the ggplot2 package v.3.3.5 [25].

### 2.4. Identification of Olfaction-Related Genes

For OBPs, CSPs, ORs, GRs, IRs and SNMPs, we created a custom nonredundant database with the putative olfaction-related genes in *A. mellifera* reported by Robertson and Wanner (2006) [26], Forêt and Maleszka (2006) [27], Forêt et al. (2007) [28], Croset et al. (2010) [29] and Nichols and Vogt (2008) [30]. Then, the BLASTX algorithm was used to identify the putative olfaction-related genes in *A. cerana* by aligning the identified olfaction-related genes in *A. mellifera* to the assembled and annotated *A. cerana* genes. These candidate olfaction-related genes in *A. cerana* were given the same names as those in *A. mellifera*. To identify the candidate ODEs, we downloaded all the candidate ODE genes of both *A. cerana* and *A. mellifera* from the NCBI database. The candidate ODEs were determined by sequence alignment to candidate ODEs of *A. mellifera* and reference to gene descriptions in *A. cerana*.

### 2.5. Real-Time Quantitative PCR

We conducted RT-qPCR to verify the expression values of the DEGs identified from the transcriptome analysis and reveal the expression profiles of selected ORs in workers and drones. Twelve DEGs, 8 upregulated and 4 downregulated genes in the drone antennae (Table S1) were selected to validate the results of DEG analysis. Six ORs (*AcOR10*, *AcOR11*, *AcOR13*, *AcOR18*, *AcOR79* and *AcOR170*) with drone-biased expression were further investigated in different tissues and individuals of different ages. The specific primers (Table S1) were designed using the Primer 3.0 plus server (https://www.ncbi.nlm.nih.gov/tools/primer-blast/index.cgi?LINK_LOC=BlastHome; accessed on 9 January 2022). Nuclear β-actin was chosen as the reference gene (Table S1). Total RNA was reverse transcribed to cDNA using the PrimeScript RT Reagent Kit with gDNA Eraser (TaKaRa, Shiga, Japan). RT-qPCR was performed with SYBR Premix Ex Taq^TM^ II (Takara, Shiga, Japan). Each reaction was performed with a total volume of 20 μL containing 2 μL of template cDNA, 10 μL of SYBR Premix Ex Taq^TM^ (2×), 0.4 μL of ROXII and 0.5 μL of each primer (10 μM). PCR was performed with a programme of 30 s at 95 °C, followed by 40 cycles of 5 s at 95 °C and 34 s at 60 °C. A melting curve was generated at 95 °C for 15 s, 60 °C for 1 min and 95 °C for 15 s. For each sample, we performed three technical replicates. Negative controls with ddH_2_O as the template were included. All reactions were performed in triplicate with an Mx3000P real-time PCR system (Stratagene, Agilent Technologies, CA, USA).

The relative expression level of these chemosensory DEGs was normalized to that of the reference gene using the 2^−ΔΔCt^ method [31]. Significant differences in expression levels were determined using one-way analysis of variance (ANOVA), and Tukey’s test was used for post hoc comparisons. All analyses were performed using SPSS 26.0 (IBM, Armonk, New York, NY, USA). The results are reported as the means ± standard errors and were plotted using GraphPad Prism 7.0 (GraphPad Software, San Diego, California, CA, USA).

## 3. Results

### 3.1. Transcriptome Sequencing Data

Six RNA-seq libraries were generated, three for *A. cerana* drones and three for *A. cerana* workers. The antennal transcriptome libraries of drones and workers yielded an average of 34,984,530 and 29,707,879 clean reads, respectively (Table 1). In each library, the Q30 was >90%, and the GC content was 36.0–40.5%. Approximately 80.34–89.63% of the clean reads were aligned to a unique location in the reference *A. cerana* genome (Table 1).

Pearson’s correlation of gene expression levels between all pairs of samples from drones and workers revealed R^2^ values > 0.99 between biological replicates and <0.74 between workers and drones (Figure S1).

### 3.2. Differentially Expressed Genes between Workers and Drones

**Figure 1:**
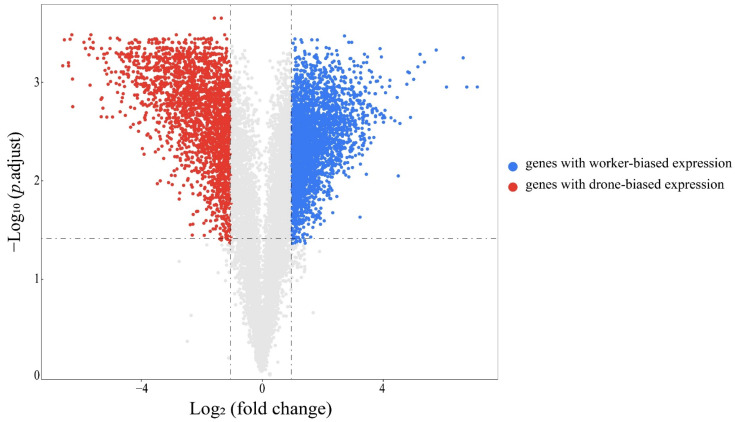
A volcano plot showing the differentially expressed genes (adjusted *p* value < 0.05 and |log_2_(fold change)| > 1, Benjamini-Hochberg method) between workers and drones. The antennae of both workers and drones included three biological replicates.

A total of 12,715 genes were detected in the antennae of *A. cerana*. There were 5262 DEGs identified between the worker antennae and the drone antennae, including 2359 genes that were expressed at higher levels in the drone antennae (genes with drone-biased expression) and 2903 genes that were expressed at higher levels in the worker antennae (genes with worker-biased expression) (Figure 1). The number of genes with drone-biased expression was significantly lower than that with worker-biased expression (χ^2^ = 70.917, *p* = 3.73 × 10^−17^).

### 3.3. GO Analysis and Pathway Analysis

**Figure 2:**
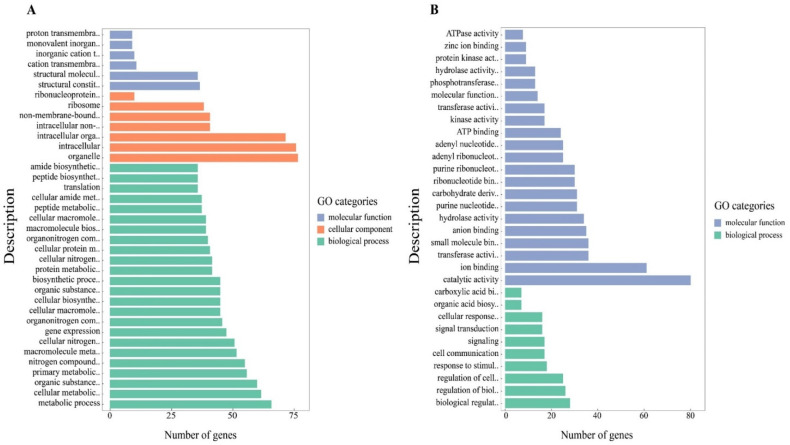
GO enrichment analysis of differentially expressed genes identified in the *Apis cerana* antennal transcriptome. (**A**) GO enrichment analysis of genes with worker-biased expression. (**B**) GO enrichment analysis of genes with drone-biased expression.

The GO enrichment analysis of the 2903 genes with worker-biased expression revealed that 37 GO terms were significantly enriched (adjusted *p* value < 0.05; Table S2). These genes were assigned to three principal categories: biological processes, molecular functions and cellular components. The genes were mainly associated with biosynthetic and metabolic processes, organelles, structural molecule activity and structural constituents of ribosomes (Figure 2A; Table S2). GO analysis of the 2359 genes with drone-biased antennal expression revealed significant enrichment for catalytic activity and binding, with assignment to only two principal categories: molecular functions and biological processes (Figure 2B; Table S2).

The KEGG pathway analysis showed that the genes with worker-biased expression were related to 5 pathways, and the most abundant groups were ribosome, spliceosome and oxidative phosphorylation (Figure S2; Table S3). In the drone antennae, the genes with biased expression were related to 25 pathways, including the Wnt signalling pathway, the MAPK signalling pathway-fly, endocytosis, the Hippo signalling pathway-fly and the FoxO signalling pathway (Figure S2; Table S3).

### 3.4. Candidate Olfaction-Related Genes and Their Expression Profiles

A total of 110 OR genes, 15 OBP genes, 5 CSP genes, 9 GR genes, 8 IR genes and 2 SNMP genes were identified in the *A*. *cerana* antennae (Table S4). We also identified 93 candidate ODEs, including 1 AOX, 4 ADs, 12 UGTs, 46 P450s, 12 GSTs and 18 esterases (Table S5). All these olfaction-related genes were expressed in each library.

**Figure 3:**
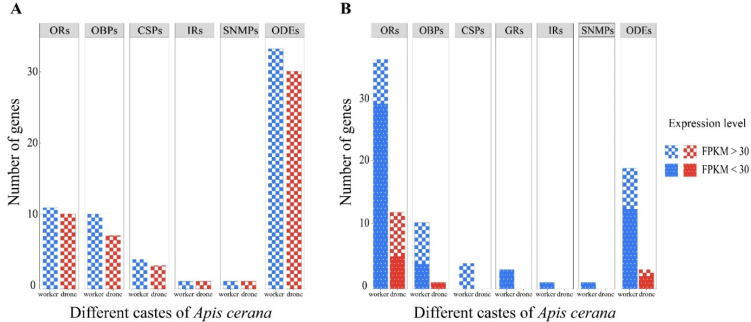
Number of olfaction-related genes in workers and drones of *A. cerana*. (**A**) Number of olfaction-related genes with high expression (FPKM > 30) identified in the *A. cerana* antennal transcriptome. (**B**) Number of differentially expressed olfaction-related genes (DEGs) with high (FPKM > 30) and low (FPKM < 30) expression identified in the *A. cerana* antennal transcriptome.

Among these 242 olfaction-related genes, a total of 17 ORs, 10 OBPs, 4 CSPs, 1 IR, 1 SNMP and 33 ODEs were highly expressed (FPKM > 30), while all the GRs exhibited relatively low expression levels (FPKM < 30) (Figure 3A; Tables S4 and S5). The numbers of ORs, OBPs, CSPs and ODEs highly expressed in drone antennae were less than those in the worker antennae, but the difference was not significant (*p* > 0.05, Fisher’s exact test) (Figure 3A; Tables S4 and S5). One IR and 1 SNMP were highly expressed and exhibited enriched expression values in both worker and drone antennae (Figure 3A; Table S4).

A total of 90 candidate olfaction-related genes showed significant differences in transcript abundance between worker and drone antennae (Figure 3B; Tables S4 and S5). A total of 36 ORs, 10 OBPs, 4 CSPs, 3 GRs, 1 IR, 1 SNMP and 19 ODEs showed worker-biased expression, while the remaining 12 ORs, 1 OBP, and 3 putative ODEs showed significantly higher expression in drone antennae (Figure 3B; Tables S4 and S5). Among these sex-biased olfaction-related genes, 7 ORs, 7 OBPs, 4 CSPs and 8 ODEs were highly expressed in worker antennae, while only 7 ORs and 1 ODE were highly expressed in drone antennae (FPKM > 30). Significantly more ORs, OBPs, GRs and ODEs had worker-biased expression (*p <* 0.05, Fisher’s exact test) (Figure 3B).

### 3.5. Validation of DEGs by RT-qPCR

We used RT-qPCR to analyse the gene expression differences between worker and drone antennae. We selected 12 DEGs (Table 2) to validate the reliability of the RNA-seq results. Eleven of these 12 genes showed significant expression differences between workers and drones (*p* < 0.05, *t* test), with 4 being upregulated in the worker antennae and 7 in the drone antennae (Table 2). The results of RT-qPCR were consistent with the results of RNA-Seq data analysis, indicating that the transcriptome analysis was reliable.

### 3.6. Expression Patterns of AcOR10, AcOR11, AcOR13, AcOR18, AcOR79 and AcOR170

**Figure 4:**
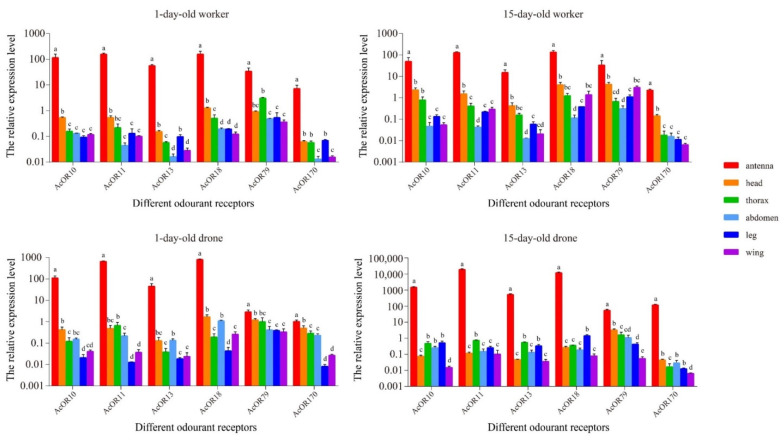
The relative expression level of *AcOR10*, *AcOR11*, *AcOR13*, *AcOR18*, *AcOR79* and *AcOR170* in different tissues of workers and drones of *A. cerana* at 1 day and 15 days of age. Data are presented as the means ± standard errors. Different lowercase letters indicate significant differences between tissues (*p* < 0.05, ANOVA).

There were clear variations in the expression levels of all 6 ORs between different tissues in differently aged workers and drones (Figure 4). Significantly different expression levels could be detected in all six tissues of workers and drones. In general, the relative expression levels of these six ORs in both workers and drones were significantly higher (*p* < 0.01, ANOVA) in the antennae than in other tissues. For *AcOR10*, *AcOR13* and *AcOR170*, the expression levels were much lower in the wings of both workers and drones at 1 day and 15 days of age. For *AcOR79* and *AcOR18*, the lowest relative expression levels were also recorded in the wings of drones at 15 days of age and in the wings of workers at 1 day of age. For *AcOR10*, *AcOR13* and *AcOR170*, significantly different (*p* < 0.05, ANOVA) expression levels were detected in the thorax of drones and workers at 15 days of age compared with 1 day of age. The relative expression levels of *AcOR10*, *AcOR18*, *AcOR79* and *AcOR170* were significantly higher (*p* < 0.05, ANOVA) in the heads of the 15-day-old workers than in those of the 1-day-old workers (Figure 4).

**Figure 5:**
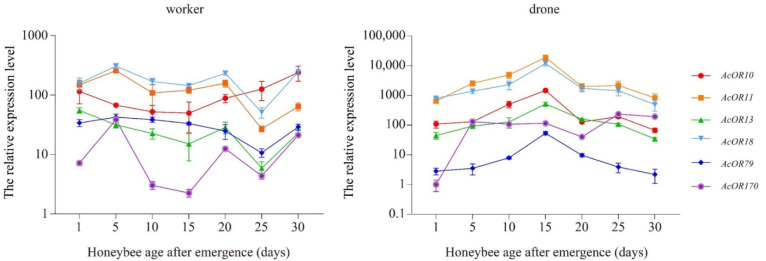
The relative expression level of *AcOR10*, *AcOR11*, *AcOR13*, *AcOR18*, *AcOR79* and *AcOR170* in workers and drones of *A. cerana* of different ages. Data are presented as the means ± standard errors.

Fluctuations in the expression levels of these six ORs were detected among differently aged workers and drones (Figure 5). With very few exceptions, the expression levels of *AcOR10*, *AcOR11*, *AcOR13*, *AcOR18* and *AcOR170* were significantly higher in the antennae of drones of different ages than in workers (*p* < 0.05, *t* test). In drones, the relative expression levels of *AcOR11* and *AcOR18* were significantly higher than relative expression levels of other ORs at all ages (*p* < 0.05, *t* test). Between workers and drones, there was no significant difference in the expression levels of *AcOR10* at 1 day, 20 days and 25 days old, *AcOR13* at 1 day and 30 days old, and *AcOR18* at 30 days old. *AcOR79* expression was significantly higher (*p* < 0.05, *t* test) in workers than in drones at all ages except 15 days. The relative expression levels of *AcOR10*, *AcOR11*, *AcOR13*, *AcOR18* and *AcOR79* peaked in drones at 15 days old, while the highest expression of *AcOR170* occurred in drones at 25 days old. *AcOR11*, *AcOR13*, *AcOR18* and *AcOR79* showed the lowest expression in workers at 25 days of age. In workers, the expression peak was detected at 1 day old (*AcOR13*), 5 days old (*AcOR11*, *AcOR18*, *AcOR79* and *AcOR170*), or 30 days old (*AcOR10*) (Figure 5).

## 4. Discussion

Chemoreception plays a fundamental role in mediating a wide range of behaviour in complex honeybee societies. *A. mellifera* and *A. cerana* are the two most closely related species in the genus *Apis*. To date, 170 ORs, 21 OBPs, 6 CSPs, 10 GRs, 10 IRs and 2 SNMPs have been annotated in the *A. mellifera* genome [26,28,29,30], and 119 ORs, 10 GR, 10 IRs [32], 15 OBPs, 6 CSPs and 2 SNMPs (reference for NCBI) have been annotated in the *A. cerana* genome. The antennal chemosensory genes expressed at different developmental stages in *A. cerana* workers have been studied using the Illumina RNA-Seq approach, and a total of 109 candidate chemosensory genes, including 74 ORs, 17 OBPs, 6 CSPs, 10 IRs and 2 SNMPs were identified [14]. In this study, we investigated the antennal transcriptome of *A. cerana* and compared the expression patterns of olfaction-related genes between workers and drones. We identified 110, 15, 5, 9, 8, 2 and 93 candidate ORs, OBPs, CSPs, GRs, IRs, SNMPs and ODEs, respectively. The difference in gene numbers identified between our study and that of Zhao et al. (2016) [14] can be explained by the clean RNA-Seq data being assembled differently. We used the reference genome of *A. cerana* [32], and Zhao et al. (2016) [14] performed de novo transcriptome assembly.

Among the 110 candidate ORs, 48 were differentially expressed between workers and drones, including 36 ORs with worker-biased expression and 12 ORs with drone-biased expression (Figure 3B). *AcOR2* was the most abundant in drone antennae, followed by *AcOR11*, *AcOR18*, *AcOR79*, *AcOR170* and *AcOR10*, with FPKM > 30 (Table S4). ORs with male-biased expression might function as pheromone receptors perceiving queen-emitted sex pheromones. The drone-biased expression of four homologous ORs (*AmOR10*, *AmOR11*, *AmOR18* and *AmOR170*) has been reported in *A. mellifera* drone antennae, and *AmOR11* was identified as the odourant receptor for the queen substance 9-oxo-2-decenoic acid (9-ODA) [33]. *A. cerana* and *A. mellifera* are the most closely related *Apis* species, and 9-ODA is the main component of sex pheromones in both species [34], so it is possible that *AcOR11* also responds specifically to 9-ODA in *A. cerana*. Compared with the results of Wanner et al. (2007) [33], we detected two additional ORs (*AcOR13* and *AcOR79*) with drone-biased expression that were expressed at high levels in drone antennae. The expression patterns in drones of different ages revealed that *AcOR10*, *AcOR11*, *AcOR13*, *AcOR18* and *AcOR79* reached a high expression peak in drones at 15 days old (Figure 5), the age when drones become sexually mature and mating occurs. Therefore, these OR types might be involved in the detection of queen-released pheromone compounds that are associated with mating behaviour. Functional characterization of these highly expressed drone-biased ORs, such as *AcOR18*, *AcOR79*, *AcOR170* and *AcOR10*, still needs to be performed.

Fifteen OBPs were detected in this study, which was consistent with the number of OBPs identified in the legs of *A. cerana* workers [15]. Most OBPs (66.7%) showed worker-biased expression, and *AcOBP1* was the most abundant in both castes (Table S4). Previous studies have also revealed that OBP1 is differentially expressed in worker and drone antennae in both *A. mellifera* and *A. cerana* [35,36]. *AcOBP1* was observed to be continuously expressed in the larval, pupal and adult stages of workers and showed detectable expression in only adult drones [35]. OBP1 can bind to the main components of the queen pheromones 9-ODA and 9-HDA (9-hydroxy-2(E)-decenoic acid) [37]. According to its expression profile in workers and drones, workers likely receive queen pheromones to regulate a number of activities throughout development, while drones detect them specifically for mating flight [35]. In addition, recent studies indicated that OBP1 could bind with some plant volatile (*β*-ionone) and larval pheromone (ethyl oleate) components, implying that OBP1 has complex physiological functions [38,39].

Only *AcOBP17* was identified as drone biased (Table S4), and previous studies have investigated its expression profiles. *AcOBP17* showed great expression changes among different developmental stages and tissues, with higher expression in the 1-day-old workers and in the thorax of 10-day-old workers [14]. It has also been reported that *AcOBP17* is expressed at significantly higher levels in the heads of *A. cerana* nurse workers challenged by *Varroa destructor* [40]. The expression of the homologous gene *AmOBP17* increased in the heads of newly emerged *A. mellifera* workers after they were injected with *Escherichia coli* [41]. It was also expressed differently in nurse bee antennae between Italian honey bees and royal jelly bees, exhibiting significantly higher expression in Italian honey bees [42]. In addition, *AmOBP17* was expressed at higher levels in the antennae of 10-day-old nurses than in those of both 10-day-old and 21-day-old foragers [43]. These studies suggested that *AmOBP17* was associated with honeybee nursing behaviour. More studies are needed to reveal its function in drones.

Five CSP genes (*AcCSP1*, *AcCSP2*, *AcCSP3*, *AcCSP4*, and *AcCSP6*) were identified in this study. These CSP genes were also identified previously in the legs of *A. cerana* workers [15]. In our study, *AcCSP6* was the least abundant and was expressed at much lower levels in both worker and drone antennae (Table S4). A previous study suggested that CSPs evolved novel nutritional functions in the ovary because high expression levels of CSP5 and CSP6 were detected in both queen and worker ovaries [44]. Three of the nine GRs and one each of the IRs and SNMPs were found to have sex-biased expression, with higher abundance in workers than in drones (Table S4), while male-biased expression of IRs has been reported in Lepidoptera [45]. For the candidate ODEs, significantly more genes were found to have worker-biased expression (Table S4). Although GRs, IRs and SNMPs have been proposed to play a role in olfactory signal recognition and ODEs play a critical role in signal chemical degradation [4], their specific functions in odourant reception in honeybees still warrant further investigation.

## 5. Conclusions

In this study, we performed transcriptome sequencing analysis on the antennae of *A. cerana* drones and workers. We identified putative olfaction-related genes and identified DEGs between workers and drones. We also investigated the expression profiles of 6 ORs in different tissues of workers and drones of different ages. Our results showed that more olfaction-related genes have worker-biased expression than drone-biased expression. The selected ORs were expressed at significantly higher levels in the antennae than in other tissues. There were clear variations in the expression levels of all 6 ORs between differently aged workers and drones. Further studies on these genes with sex-biased expression are needed to reveal their specific functions in odourant reception in different honeybee castes. The results will contribute to future research on the olfactory mechanism of *A. cerana* and will help better reveal the odourant reception variations between different biological castes of honeybees.

## Figures and Tables

**Table 1:** Summary of the *Apis cerana* antennal transcriptome sequencing data.

Sample Name	Raw Reads	Clean Reads	Mapping to Reference Genome			Q30	GC Content
			Total Mapped Ratio	Uniquely Mapped Ratio	Multiple Mapped Ratio		
Ac_drone1	38,909,258	37,006,285	88.71	87.04	1.67	93.98%	39.50%
Ac_drone2	35,430,933	33,495,876	82.02	80.34	1.68	93.98%	40.50%
Ac_drone3	36,043,980	34,451,429	88.06	86.14	1.92	94.21%	38.00%
Ac_worker1	31,093,034	28,795,612	91.77	89.63	2.14	94.04%	37.00%
Ac_worker2	31,606,090	29,115,010	91.91	89.71	2.20	94.04%	36.00%
Ac_worker3	34,306,996	31,213,014	90.92	88.65	2.27	93.45%	36.00%

**Table 2:** Comparative RNA-Seq and real-time quantitative PCR (RT-qPCR) results for twelve olfaction-related genes.

Gene Name	Log_2_ (Fold Change) Worker vs. Drone	
	RNA-Seq	RT-qPCR
*AcOR10*	−1.9696	−2.3067
*AcOR11*	−3.7412	−3.6056
*AcOR13*	−1.5385	0.2311
*AcOR18*	−2.2875	−2.4844
*AcOR79*	−2.4173	−2.8022
*AcOR170*	−3.4653	−3.6911
*AcOBP1*	1.3586	1.0198
*AcOBP2*	4.213	3.4844
*AcOBP17*	−1.7026	−2.9178
*AcCSP1*	3.803	2.3789
*AcCSP3*	2.2829	2.1622
*Ac_venom carboxylesterase-6*	−1.3597	−2.6311

## Data Availability

The RNA-seq data of *A. cerana* in this study have been submitted to the NCBI Sequence Read Archive (accession number PRJNA843558).

## Supplementary Materials

The following supporting information can be downloaded at https://www.mdpi.com/article/10.3390/genes13101771/s1. Figure S1: Pearson correlation coefficients of global expression values between samples; Figure S2: KEGG pathway analysis of differentially expressed genes identified in the *A. cerana* antennal transcriptome. (A) KEGG pathway analysis of genes with worker-biased expression. (B) KEGG pathway analysis of genes with drone-biased expression; Table S1: Primers used for RT-qPCR analysis; Table S2: GO enrichment analysis of differentially expressed genes identified in *A. cerana* antennae; Table S3: KEGG pathway analysis of differentially expressed genes identified in *A. cerana* antennae; Table S4: Candidate ORs, OBPs, CSPs, GRs, IRs and SNMPs identified in *A. cerana* antennae; Table S5: Candidate ODEs identified in *A. cerana* antennae.
