# Supplementary figures and images for: Sex-Biased Expression of Olfaction-Related Genes in the Antennae of Apis cerana (Hymenoptera: Apidae)
Source: Genes (Basel). 2022 Sep 30;13(10):1771. doi: 10.3390/genes13101771 (PMC9602017; doi:10.3390/genes13101771)

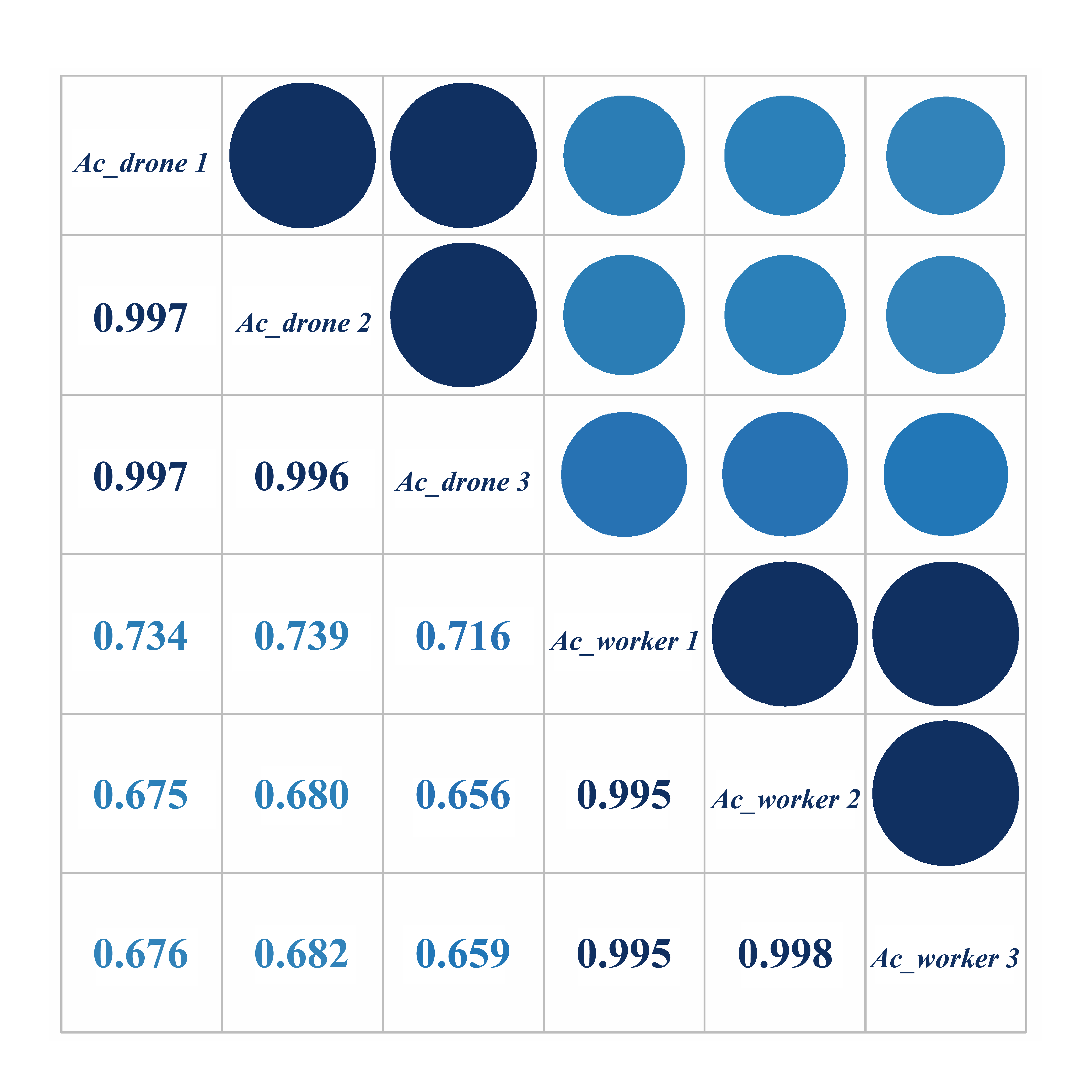

Supplement: Supplementary file 1 [file genes-13-01771-s001.zip › Figure S1 .jpg]

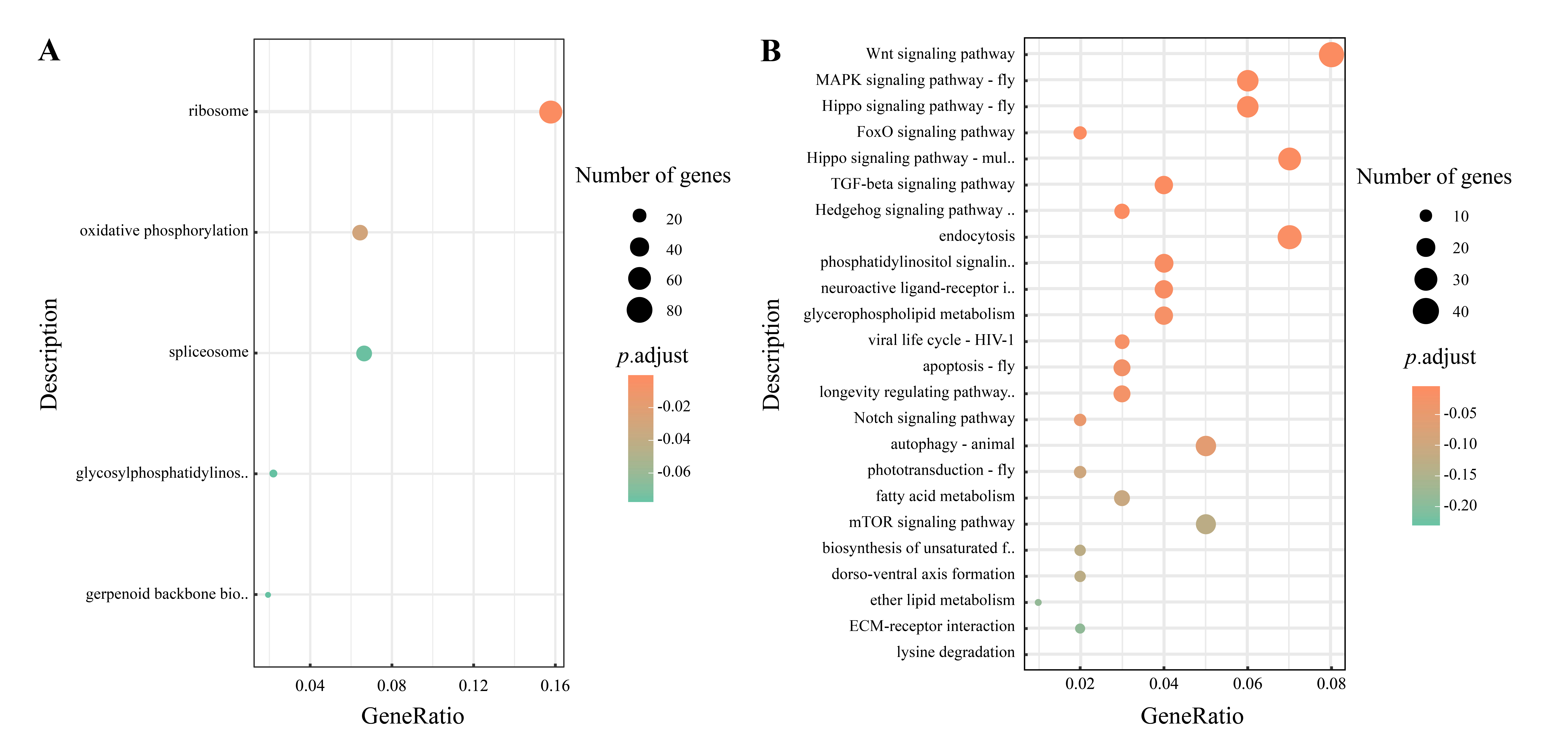

Supplement: Supplementary file 1 [file genes-13-01771-s001.zip › Figure S2 .jpg]
